# Yield, Nutritional Composition, and Digestibility of Conventional and Brown Midrib (BMR) Pearl Millet as Affected by Planting and Harvesting Dates and Interseeded Cowpea

**DOI:** 10.3390/ani13020260

**Published:** 2023-01-12

**Authors:** Madeline Oskey, Cesar Velasquez, Omar Manuel Peña, John Andrae, William Bridges, Gonzalo Ferreira, Matias Jose Aguerre

**Affiliations:** 1 Department of Animal and Veterinary Sciences, Clemson University, Clemson, SC 29634, USA; 2 Department of Plant and Environmental Sciences, Clemson University, Clemson, SC 29634, USA; 3 Department of Mathematical and Statistical Sciences, Clemson University, Clemson, SC 29634, USA; 4 School of Animal Sciences, Virginia Tech, Blacksburg, VA 24061, USA

**Keywords:** digestibility, summer annuals, maturity, cowpea

## Abstract

**Simple Summary:**

A major challenge for farming systems is the seasonality of forage production, in particular, the quantity and quality gap that usually occurs during mid to late summer. Pearl millet is a drought-tolerant, warm-season annual crop that adapts well to relatively infertile and acidic soils and can potentially be mixed with summer annual legumes. In this study, we evaluated in field plots the effect of conventional and brown midrib (BMR) pearl millet on yield, nutritional composition, and fiber digestibility when planted at different dates, harvested at different maturity stages, and when mixed with cowpea. We determined the degradability of neutral detergent fiber under in vitro conditions (IVNDFD) using rumen fluid from lactating dairy cows. The results of this study showed that the BMR pearl millet had a consistently higher IVNDFD compared to a conventional genotype. Delaying pearl millet planting had a significant impact on forage yield. Under the conditions of this study, mixing cowpea with pearl millet had little effect on quality but penalized forage yield.

**Abstract:**

The objective of this study was to evaluate the yield, nutritional composition, and digestibility of conventional (CON) and brown midrib (BMR) pearl millet (PM) with different establishment dates, maturity at harvest and when mixed with cowpea (CWP). In trial 1, CON and BMR were planted on two different dates. In trial 2, CON and BMR, mixed or not with CWP, were harvested when PM was at the boot or heading stages. In trial 1, dry matter (DM) yield was similar between both PM genotypes but delaying establishment reduced DM yield by 30%. Additionally, BMR had a lower concentration of acid detergent lignin (ADL) and a higher in vitro neutral detergent fiber digestibility (IVNDFD) compared to CON. In Trial 2, the DM yield was 7.3% higher for CON compared to BMR, and PM with the BMR trait had a lower level of ADL and higher IVNDFD compared to CON. Mixing PM with CWP had negligible effects on nutritional composition but reduced DM yield by 8.3%. Results of these studies indicated that fiber from BMR PM is more digestible than CON but, in one of the trials, this occurred at the expense of lower DM yield. Mixing CWP with PM negatively impacted DM yield.

## 1. Introduction

A major challenge of farming systems depending on cool- and warm-season perennial grasses is the seasonality of forage production. In particular, there is a quantity and quality gap that usually occurs during mid to late summer [1]. Summer annual grasses can complement perennial forage systems by increasing the number of grazing days in beef production systems [1]. In addition, annual summer grasses are an alternative forage for dairy farming systems. Although they may not replace corn silage as the standard in dairy diets, summer annual grasses can complement corn in regions prone to short-term drought stress, grow better on soils of marginal suitability for corn silage production, or be an emergency crop for late plantings destined for silage [2,3].

Pearl millet (*Pennisetum glaucum*) is a drought-tolerant, warm-season annual crop that adapts well to relatively infertile and acidic soils and can potentially be mixed with summer annual legumes [4,5]. Although several varieties containing the brown midrib (BMR-low lignin) trait are available, data on forage yield and quality under different management practices are lacking. For example, although staggered plantings of pearl millet are usually recommended to better distribute forage production throughout the growing season, later planting can reduce yield potential [6]. In addition, a major factor affecting NDF digestibility (NDFD) is the maturity stage at which the forage is harvested. As plants mature, there is a concomitant increase in biomass yield and a reduction in fiber digestibility due to the increase in cross-linkages between lignin with other cell wall components [7]. Although both management tools are frequently used by farmers to maximize both yield and quality, few studies [8,9] have been conducted to compare the impact of planting date and maturity stage at harvest between conventional and BMR pearl millet genotypes.

Fertilization with high levels of nitrogen (N) is usually required to maximize pearl millet forage production, which can increase the cost of forage production. Legume species can fix N from the atmosphere through a symbiotic relationship between plants and bacteria in the soil [10], and mixing grasses with legumes can increase the crude protein (CP) concentration of the forage [11]. Vine climbing legumes like cowpea (*Vigna unguiculata*) can tolerate some shade and fill-in rows in stands of summer annual grasses. In addition, cowpea is a viable option to increase CP concentration in forage rather than purchasing costly protein supplements [12]. Thus, interseeding summer annual grasses with legumes like pearl millet and cowpea, respectively, have the potential to provide high forage quality, increase residual soil N, improve biomass yields of the successive winter crops, and reduce fertilizer cost.

We hypothesized that pearl millet containing the BMR trait will increase forage quality, especially fiber digestibility, relative to conventional pearl millet, and that biomass yield can be maximized by manipulating planting date and maturity stage at harvest. We also hypothesized that mixing cowpea with pearl millet would increase CP concentration and NDFD without affecting forage yield. Thus, the objectives of these studies were to evaluate the effect of conventional and BMR pearl millet on forage yield, nutritional composition, and NDFD when planted at different dates and harvested at different maturity stages. An additional objective was to evaluate the impact of mixing pearl millet with cowpea on yield and forage quality.

## 2. Materials and Methods

### 2.1. Experimental Sites and Climate Data

Two studies were conducted at the Simpson Research Farm, Clemson University, located in Pendleton, South Carolina (34°37′38.0″ N 82°43′33.5″ W) during the warm growing season (April to October) of 2018 and 2019. Weather data were collected from a weather station at the research site (Onset HOBO RX3000 Cellular Weather Station, Bourne, MA). Historic weather data (1981 to 2010) were collected from a weather station located at Sandy Springs, SC, using the National Centers for Environmental Information of the National Oceanic and Atmospheric Administration (NOAA, US Department of Commerce). The soil type for trial 1 (2018) was an Appling sandy loam with 2 to 6% slopes (ApB), whereas the soil type for Trial 2 (2019) was Cecil sandy loam with 2 to 6% slopes (CdB).

### 2.2. Experimental Design

Trial 1. Conventional (CON; Tifleaf-3; Hancock Seed & Co., Dade City, FL, USA) and brown midrib (BMR; Exceed; Coffey Forage Seeds, Plainview, TX, USA) pearl millet varieties were planted at a seeding rate of 28 kg/ha of pure live seeds (PLS) on May 9 and 18, 2018 (Early and Late, respectively). The trial was designed as a randomized complete block design with a 2 × 2 factorial arrangement of treatments and 5 replications (i.e., blocks). Within each of the 5 blocks, 2 plots (1.5 m × 6.1 m) were planted with one of the 2 pearl millet varieties, one for each planting date, using a 7-row drill equipped with an Almaco cone. Fertilizer was applied to each plot before planting (22 kg N/ha, 56 kg P_2_O_5_/ha, and 45 kg K_2_O/ha) according to recommendations after soil analysis, and after each harvest (23 kg N/ha). The plots were harvested when the crop reached the early heading stage of maturity (3 harvests total).

Trial 2. Conventional (CON; “Tifleaf-3”) and brown midrib (BMR; “Exceed”) pearl millet varieties were planted in monoculture (28 kg PLS/ha) or in a mixture (14 and 28 kg PLS/ha, respectively) with cowpea (CWP; “Iron and Clay”; Hancock Seed & Co., Dade City, FL, USA) on May 18, 2019. The trial was designed as a randomized complete block design with a 2 × 2 × 2 factorial arrangement of treatments and 4 replications (i.e., blocks). Within each of the 4 blocks, 2 plots (1.5 m × 6.1 m) were planted with one of the 4 forage treatments. One of the 2 plots within the same block was harvested when the pearl millet crop reached the boot stage of maturity, and the other one at the heading stage of maturity (2 harvests at each maturity stage). Plots were planted using a 7-row plot drill equipped with an Almaco cone. Fertilizer was applied to each plot before planting (22 kg N/ha, 56 kg P_2_O_5_/ha, and 45 kg K_2_O/ha) according to recommendations after soil analysis, and after each harvest (23 kg N/ha).

### 2.3. Sample Collection and Analyses

In both trials, the forage biomass of each plot was harvested using a Carter plot forage harvester (Carter Manufacturing Co., Brookston, IN, USA). After weighing the harvested biomass, samples from each plot were collected in plastic bags, immediately placed in a cooler with ice, and transferred to the laboratory for storage at −20 °C. Samples were thawed and dried at 55 °C in a forced-air oven for 48 h. The resulting dry matter (DM) concentration was used to estimate DM yield (kg/ha). Dried samples were ground to pass through a 1-mm screen of a Wiley mill (Arthur H. Thomas, Philadelphia, PA, USA). Ground samples were dried at 105 °C for 24 h to determine analytical DM. Ash concentration was determined after combusting samples in a furnace for 3 h at 600 °C (Method 942.05, AOAC) [13]. For each sample, a subsample was separated and submitted to Cumberland Valley Analytical Services (Waynesboro, PA, USA) to determine the concentrations of N (Method 990.03, AOAC) [14] and water-soluble carbohydrates as described by Hall et al. [15]. Crude protein concentration was calculated as percentage N × 6.25 after combustion analysis. Neutral detergent fiber (aNDFom) and ADFom concentrations were determined using an Ankom200 Fiber Analyzer (Ankom Technology, Faiport, NY, USA) and corrected for ash concentration. Sodium sulfite and α-amylase (Sigma no. A3306: Sigma Chemical Co., St. Louis, MO, USA) were included for the NDF analysis [16]. After determining the ADF, the fiber residue was incubated for 3 h in 72% sulfuric acid within 4 L jars that were placed in a Daisy II Incubator (Ankom Technology) for ADL determination.

Care and handling of animals used for collecting rumen contents and in situ incubations was conducted as outlined in the guidelines of the Clemson University Committee on Animal Use (AUP2019-074). In vitro DM digestibility (IVDMD), in vitro true DM digestibility (IVTDMD), and in vitro NDF digestibility (IVNDFD) were determined using a Daisy II rotating-jar in vitro incubator (Ankom Technology). Samples were incubated for 30 h following the procedures described by Ferreira and Mertens [17]. A composite inoculum was prepared with rumen fluid and solids collected before the morning feeding from two ruminally fistulated lactating dairy cows that were fed a diet containing 44.0% corn silage, 4.1% triticale, and 51.9% concentrate mix (DM basis). To determine undegraded NDF (uNDF), 0.25 g of sample was weighed into F57 Ankom bags (Ankom Technologies) and incubated in the rumen of 2 ruminally fistulated and multiparous cows (1 Jersey and 1 Holstein) for 240 h. The cows were fed the same diet described above. After the 240 h incubation, bags were weighed and subjected to NDF analysis as described previously. Harvested yield of potentially digestible NDF (pdNDF, kg/ha) was calculated by multiplying the concentration of the pdNDF by the DM yield of the corresponding plot.

### 2.4. Statistical Analysis

Data were analyzed with the mixed procedure of SAS (SAS version 9.4, SAS Institute Inc., Cary, NC, USA). For Trial 1, the statistical model included the random effect of the block (n = 5); the fixed effect of planting date (n = 2); the fixed effect of pearl millet genotype (n = 2); the fixed effect of harvest (n = 2) as a repeated measure; the interaction between planting date and pearl millet genotype; the interaction between genotype and harvest; the interaction between planting date and harvest; the interaction between genotype, planting date and harvest; and the residual error. The first-order autoregressive covariance structure was used to fit a time-series-type covariance structure in which the correlation declines as a function of time.

For trial 2, the statistical model included the random effect of the block (n = 4), the fixed effect of maturity at harvest (n = 2), the fixed effect of pearl millet genotype (n = 2), the fixed effect of cowpea (n = 2), the interaction between pearl millet genotype and cowpea, the fixed effect of harvest (n = 2), all possible interactions, and the residual error. Significant differences and tendencies to differ were declared at *p* < 0.05 and *p* ≤ 0.10, respectively.

## 3. Results

### 3.1. Weather Conditions

**Figure 1:**
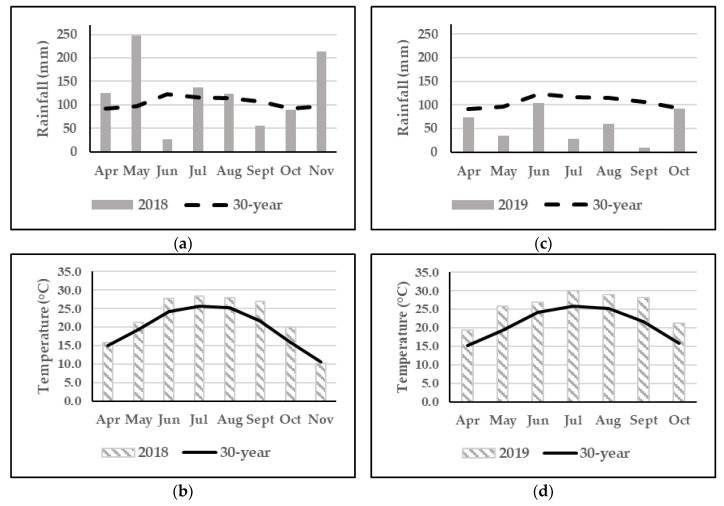
Total monthly precipitation (mm) and mean monthly temperature (°C) and 30-yr historical average for Sandy Springs, SC, during the 2018 (**a**,**b**) and 2019 (**c**,**d**) growing seasons.

Rainfall during the 2018 growing season was above the 30-year mean during the spring (except for June) and similar to historic averages for most of summer and fall (Figure 1a). Temperatures during the 2018 growing season were higher than the 30-year mean (Figure 1b). Rainfall was below the 30-year mean during most of the 2019 growing season, with distinctly dry conditions during May, July, August, and September (Figure 1c). The temperatures during the 2019 growing season were higher than the 30-year mean (Figure 1d).

### 3.2. Forage Yield, Chemical Composition, and In Vitro Digestibility

Trial 1. Table 1 presents the least squares means on the effects of pearl millet genotype and planting date on biomass yield and forage chemical composition. No interaction between genotype and planting date was observed on the reported variables. Throughout the growing season, CON and BMR genotypes yielded similar biomass (10,033 kg/ha of DM). In addition, CON and BMR genotypes had similar concentrations of ash (10.7% of DM), CP (15.9% of DM), aNDFom (57.5% of DM), ADFom (33.2% of DM), and WSC (7.0% of DM). However, compared to BMR pearl millet, CON had a greater concentration of ADL on a DM-basis (3.35 vs. 3.74% of DM) and on an NDF-basis (6.55 vs. 5.82% of NDF). Planting pearl millet 14 d earlier resulted in a 30% increase of biomass yield (11,342 vs. 8725 kg/ha), and this increase occurred regardless of pearl millet genotype. Planting date did not affect the concentrations of ash, CP, NDF, ADF and ADL; however, pearl millet planted early had a greater concentration of WSC than pearl millet planted 14 d later (7.28 vs. 6.81 % DM).

**Figure 2:**
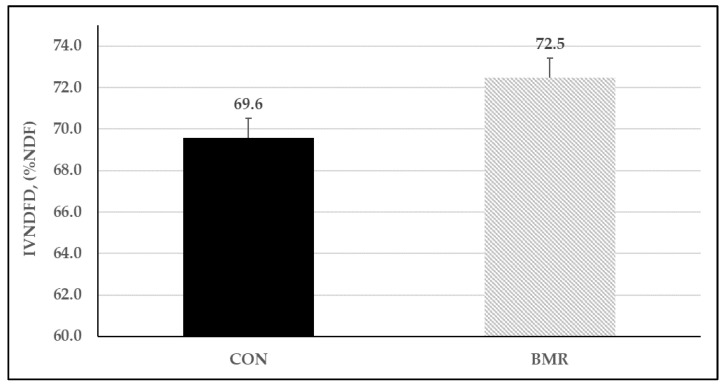
In vitro NDF digestibility (IVNDFD) of pearl millet genotypes planted at two different dates (May 9th and May 18th) and harvested three times during 2018 (Trial 1). Gen (*p* < 0.01), planting date (*p* = 0.60), and Gen × planting date (*p* = 0.28); vertical bars indicate standard errors of the mean.

**Figure 3:**
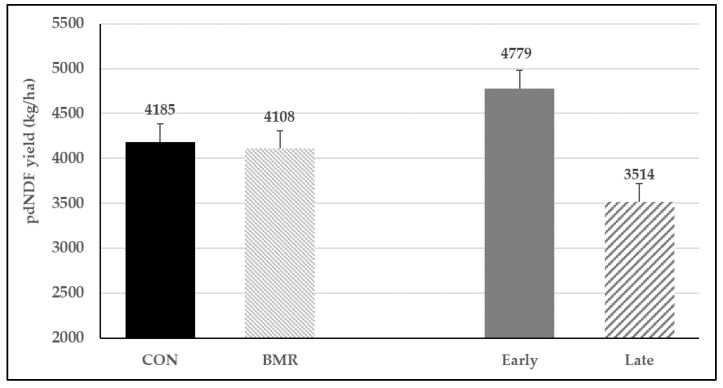
Potentially digestible NDF (pdNDF) yield per ha for Trial 1. Gen (*p* = 0.75), planting date (*p* < 0.01), and Gen × planting date (*p* = 0.52); vertical bars indicate standard errors of the mean.

The concentrations of uNDF and pdNDF differed between pearl millet genotypes (Table 2). On an NDF-basis, BMR pearl millet had the least concentration of uNDF compared to CON (28.4 vs. 32.0% aNDFom). Thus, pearl millet with the BMR genotypes had a higher concentration of pdNDF (71.6 vs. 68.0% of NDF) than conventional pearl millet. Planting date did not affect uNDF and pdNDF concentrations nor IVTDMD and IVNDFD. In addition, although the BMR genotype had similar IVDMD and IVTDMD than the CON genotype (Table 2), the BMR genotype had greater IVNDFD than the CON (Figure 2). The yield of pdNDF did not differ between BMR and CON pearl millet, but due to the increase of DM yield, pdNDF yield (kg/ha) was 36% higher when pearl millet was planted earlier in the season (Figure 3).

We observed a significant effect (*p* < 0.01) of harvest time on DM yield. The majority of the harvested biomass DM was obtained in the first and second harvest (58 and 33%, respectively) with a minor proportion (9%) of the total biomass yield collected in the last harvest of the growing season. Chemical composition was similar between the first and second harvest. However, relative to the first two harvests, the concentrations of ash, CP, and ADL were higher and the concentrations of aNDFom and ADFom were lower in the last harvest. In addition, the first harvest had the lowest (67.5%), the second harvest had the intermediate (70.7%), and last harvest had the highest (75.0%) IVNDFD (*p* < 0.01). An interaction existed between genotype and harvest time for ADL (*p* < 0.01) and IVNDFD (*p* < 0.01). The BMR genotype had less ADL and ADL as a percentage of aNDFom concentration than the CON genotype in the first and second harvests but not in the last one, and IVNDFD followed the same response pattern.

Trial 2. Table 3 presents the least squares means for the effects of pearl millet genotypes grown in monoculture or in mixture with CWP at different maturity stages at harvest on biomass yield and forage nutritional composition. No interaction between pearl millet genotype, CWP, and maturity stage at harvest existed for the reported variables. The CON genotype yielded 8.3% more biomass than the BMR genotype (8052 vs. 7382 kg/ha, respectively). In addition, including CWP in the forage mix reduced biomass yield by 8.3% for both pearl millet genotypes.

Pearl millet genotype did not affect the concentrations of DM (28.6%), ash (14.2% DM), CP (14.7% DM), and WSC (8.48% DM). However, compared to BMR pearl millet, CON had a higher concentration of aNDFom (54.4 vs. 52.7% DM), ADFom (36.7 vs. 34.8% DM) and ADL (4.80 vs. 4.38% DM) at harvest. The concentration of ADL as a proportion of aNDFom did not differ between genotypes.

Regardless of the genotype, growing pearl millet in mixture with CWP increased the concentration of CP (15.3 vs. 14.2% DM) and reduced the concentration of aNDFom (54.4 vs. 52.7% DM) of the forage, but had negligible effects on all other measured nutrients. Pearl millet harvested at the heading stage resulted in a numerical increase in biomass yield compared to forage harvested at the boot stage (7927 vs. 7508 kg/ha, respectively). In addition, we observed an interaction (*p* < 0.01) between maturity stage and when the forage was harvested (cut number). Dry matter yield was higher when the forage was harvested at the heading stage compared to the boot stage in the first cut (6735 vs. 5854 kg/ha), but the more mature forage had a lower yield in the second cut (1191 vs. 1652 kg/ha). Pearl millet maturity stage at harvest had no effect on nutrient content (Table 3) except for a small increase in DM (28.1 vs. 29.3%, *p* = 0.02) and ash (13.6 vs. 14.9% DM, *p* < 0.01) concentrations on the more mature forage. The highest proportion of harvested biomass was obtained in the first harvest (82%), with a smaller proportion of the total biomass yield collected in the last harvest of the growing season (18%). Compared with the first harvest, CP and ADL were higher while aNDFom and WSC were lowest in the second harvest.

**Figure 4:**
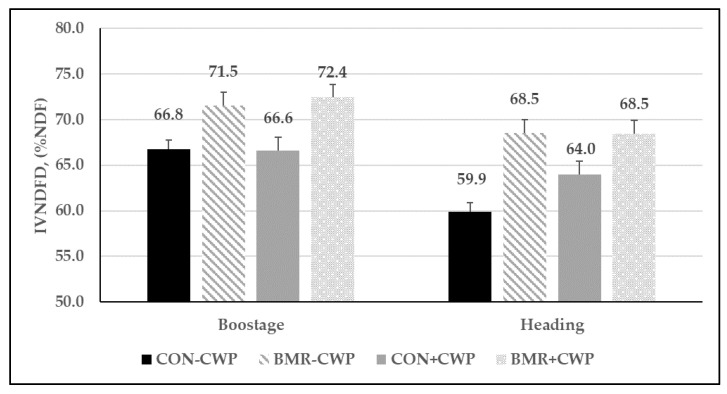
In vitro NDF digestibility (IVNDFD) of pearl millet genotypes mixed or not with cowpea and harvested at boot or heading maturity stages in 2019 (Trial 2). Gen (*p* < 0.01), CWP (*p* = 0.22), maturity (*p* < 0.01), and Gen × CWP (*p* = 0.43); vertical bars indicate standard errors of the mean.

The concentration of uNDF and pdNDF differed between the pearl millet genotypes (Table 4). On an aNDFom basis, BMR pearl millet had the least concentration of uNDF compared to CON (29.9 vs. 34.2%). Thus, pearl millet with the BMR genotype had a higher concentration of pdNDF (70.1 vs. 65.7% of aNDFom) than CON pearl millet. Compared with CON, the BMR genotype had a higher IVDMD (72.3 vs. 69.1%), IVDMTD (80.1 vs. 76.7%) and IVNDFD (70.2 vs. 64.3%, Figure 4). Mixing CWP with pearl millet had no impact on uNDF, pdNDF, IVDMD, and IVNDFD. The forage harvested at the boot stage had the lowest concentration of uNDF (29.9 vs. 34.2% of aNDFom) and higher coefficients of IVDMD (72.3 vs. 69.1%) and IVDMTD (80.0 vs. 77.0%) compared with the more mature forage (Table 4). Furthermore, the forage harvested when PM was at the boot stage had a higher IVNDFD (69.4 vs. 65.2%) compared with the PM harvest at the heading stage (Figure 4). Compared with the first harvest, uNDF was lower (27.8 vs. 36.4% of aNDFom) and pdNDF was higher (72.2 vs. 63.6% of aNDFom) in the second harvest. However, IVNDFD was similar between both harvests.

**Figure 5:**
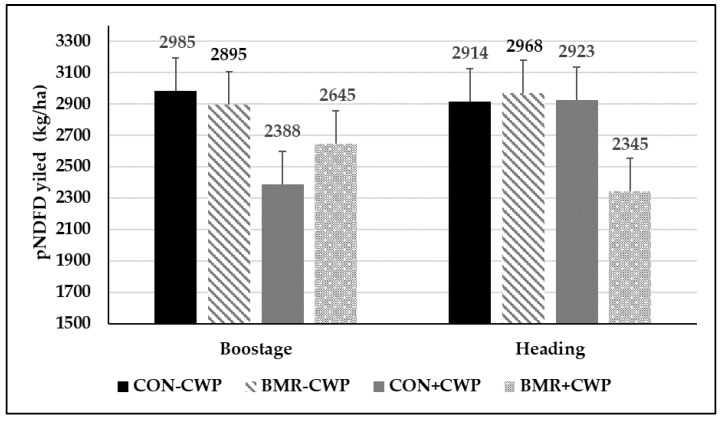
Potentially digestible NDF (pdNDF) yield per ha for Trial 2; vertical bars indicate standard errors of the mean.

The yield of pdNDF was not different between BMR and CON pearl millet, but when CWP was added to both pearl millet genotypes, pdNDF yield was reduced by 12.4% (Figure 5). As a result of the higher concentration of pdNDF (NDF-basis) for forage harvested at the boot stage, pdNDF yield (kg/ha) was not different between both maturity stages (2770 ± 402, Figure 5).

## 4. Discussion

### 4.1. Effect of Genotype and Legume Mix

Under the conditions of this study, the DM yield of BMR PM was numerically (trial 1) or tended (trial 2) to be lower than the CON PM, suggesting that PM with the BMR trait could have a negative impact on biomass yield. The lower DM yields of the BMR trait compared with CON hybrids has been previously documented in corn silage [18,19]. In addition, several authors [8,9,20] observed a consistent reduction in DM yield ranging between 23 and 41% when comparing a BMR with a non-BMR pearl millet genotype. However, it is important to highlight the small number of PM varieties with the BMR trait that had been previously evaluated and with few replications over several seasons and growing conditions, which warrants further studies to confirm the impact of the BMR trait on forage yield. In both trials reported in this study, when yield was measured as pdNDF (kg/ha), the higher fiber digestibility of the BMR counteracted the higher DM yield of the CON genotype.

Chemical composition (ash, CP, fiber fractions and WSC) of PM varieties reported in both trials was within the expected range for this annual grass when grown under similar conditions [21,22]. In line with the observations of Ferreira et al., BMR PM had a consistently lower ADL concentration (aNDFom-basis) and uNDF than non-BMR pearl millet [23]. In both of the current trials, lower ADL content for the BMR trait increased fiber digestibility by 6.5%. Similarly, Cherney et al. reported a 5.5% increase in NDF digestibility when wethers were fed BMR PM compared with non-BMR PM [24]. Interestingly, the improvement in NDF digestibility observed in the current study, when the BMR trait was present, is smaller than the one observed in other annual summer grasses. Oba and Allen [25,26] observed a 22.4% increase in NDFD between conventional and BMR corn silages. Similarly, Sanchez-Duarte et al. reported a 14.5% increase in NDFD between conventional and BMR genotypes of sorghum silages [27]. The wide range in reported NDFD differences between BMR and non-BMR grasses highlights the importance of measuring forage ADL concentration, on an aNDFom-basis, when evaluating fiber digestibility. Oba and Allen and Sanchez Duarte et al. observed a 23 to 30% reduction in ADL content (NDF-basis) between non-BMR and BMR genotypes [25,26,27]. However, in both the current trials, the smaller difference on NDFD was consistent with the much lower reduction in ADL concentration (aNDFom-basis) between the two PM varieties (11 and 6% for trial 1 and 2, respectively). These results agree with the findings of Ferreira et al., suggesting that the level of response to forage digestibility due to the BMR mutation is influenced by forage species [23].

In the current study, intercropping PM with cowpea reduced the DM yield by 8.3%. Bryan and Materu did not observe an impact on forage DM yield when interseeded cowpea and corn were compared with a corn monoculture [28]. Similarly, Armstrong et al. observed similar yields when corn was grown alone or mixed with lablab or velvet bean [12]. Cowpea has a lower DM content compared to pearl millet, and with a lower plant population of the grass in the forage mix compared to the monoculture treatment, the overall DM yield per ha was likely penalized. Furthermore, cowpea regrowth is poor, resulting in empty spaces that will not produce forage or that can be filled by undesired weeds after the first harvest. Thus, cowpea may be a better option for forage mixes with one-cut forages like sorghum or corn. Finally, drought conditions during the growing season might have further penalized cowpea regrowth potential.

Although it did not have an effect on the overall forage digestibility, the results of this study support our hypothesis that PM and CWP grown together can increase the CP content of the forage mix compared to the monoculture of PM. The 7.4% increase in CP concentration observed in trial 2 is lower than the 9 to 16% increase in CP reported by Bryan and Materu and Armstrong et al. when intercropping corn with several annual legumes [12,28]. This statistically, albeit negligible, increase in CP content observed in the current study will likely have a minor impact on animal response for most livestock species. Although it could help reduce expensive protein supplementation in dairy cow diets, producers will probably need to grow these in a larger land area to compensate for the reduction in DM yield. Future research should also investigate the potential benefits of the residual effects of N fixation by cowpea on the follow-up crop.

### 4.2. Effect of Planting Date and Maturity Stage at Harvest

Hancock and Durham observed that DM yields were highest when pearl millet was planted in late April and decreased linearly (by as much as 90 kg/ha) for each day planting was delayed [6]. In the current study, delayed planting by 14 d resulted in a decreased yield of 187 kg/ha. The large discrepancy between studies is likely associated with differences in weather conditions during the experiments and the yield potential of the tested genotypes. In addition, Hancock and Durham replicated the study over 3 years, compared with our single year study [6]. However, the results of both studies clearly illustrated the great likelihood of a DM yield reduction associated with late planting of pearl millet. Previous studies with corn silage have shown a negative impact on forage quality as planting dates progressed through the growing season [29]. However, under the conditions of this study, a delayed planting date had minimal impact on forage quality. Estimating forage yield per ha on a pdNDF-basis highlighted the potential impact of a lower DM yield due to late planting on milk or meat production per ha, despite the similar pdNDF yield observed between both pearl millet varieties (Figure 4).

As expected, forage yield in trial 2 was higher when forage was harvested at a more advanced stage of maturity, but only in the first cutting of the season. This yield advantage obtained by delaying harvest was mostly offset in the second cut. Precipitation after the first harvest may have affected plant regrowth. The amount of recorded rain was higher between the first and second cut of forage harvested at the boot stage (100 mm) compared with forage harvested at the heading stage (56 mm). Thus, future research should evaluate the effects of pearl millet maturity at harvest under non-drought conditions, such as those observed in trial 2, in order to remove the impact of water stress on yield. Interestingly, PM with the BMR trait and harvested at the heading stage had a similar yield (Table 3) and slightly higher IVNDFD (Figure 4) compared with CON pearl millet harvest at the boot stage. According to these results, planting BMR pearl millet instead of non-BMR will increase management flexibility by allowing a wider optimal harvest window and potentially reducing the number of cuts per season while still producing the same amount of forage, and with similar fiber digestibility. Using the pdNDF yield to compare the two PM varieties (Figure 5) supports this assumption that mature (heading stage) BMR will have the same animal production potential as early harvested CON pearl millet (2657 vs. 2687 kg/ha of pdNDF, respectively). Therefore, indicators that include both DM yield and fiber digestibility should be considered when selecting PM varieties (and other annual summer grasses) that contain the BMR trait for a forage program. Furthermore, estimating the pdNDF per hectare allows producers to visualize the potential impact of different management tools, such as maturity at harvest, on livestock performance.

## 5. Conclusions

The pearl millet hybrid evaluated in this study that contains the BMR trait had a consistently higher digestibility of aNDFom compared with a conventional genotype, which will allow producers to obtain a higher quality forage when these two varieties are harvested at a similar maturity stage. From a management standpoint, harvest timing of BMR pearl millet would appear to be more flexible by allowing forage harvest to be delayed until a more advanced stage of maturity without penalizing forage quality as much as a conventional pearl millet. However, while it did not affect forage quality, a 30% reduction in DM yield was associated with a 14-day delay in planting. In addition, planting a mix of pearl millet and cowpea increased CP concentration, but at the expense of a lower biomass yield, which will result in more land area needed to get the same amount of forage as in a pearl millet monoculture. Future work should be conducted to further investigate the effect of pearl millet with the BMR trait when it is included in the forage program of dairy or beef cattle operations.

## Figures and Tables

**Table 1:** Yield and nutritional composition of pearl millet genotypes planted at different dates (Trial 1).

		PM Genotype ^1^			*p*-Value ^2^		
Item	Planting	CON	BMR	SEM	Gen	Planting	Gen × Planting
DM yield, kg/ha	Early	11,637	11,047	597	0.14	<0.01	0.58
	Late	9348	8100				
DM, %	Early	23.6	22.7	0.52	0.40	0.01	0.34
	Late	21.6	21.6				
Ash, % DM	Early	11.8	12.3	0.49	0.29	0.41	0.06
	Late	12.4	10.9				
CP, % DM	Early	15.8	15.8	0.46	0.98	0.53	0.98
	Late	16.0	16.0				
aNDFom, % DM	Early	57.3	57.6	0.80	0.25	0.71	0.43
	Late	56.3	57.9				
ADFom, % DM	Early	33.0	32.7	0.52	0.53	0.17	0.23
	Late	33.1	34.1				
ADL, % DM	Early	3.62	3.19	0.16	0.03	0.11	0.82
	Late	3.86	3.51				
ADL, g/kg aNDFom	Early	6.30	5.60	0.28	0.02	0.11	0.93
	Late	6.80	6.05				
WSC, g/kg DM	Early	7.33	7.22	0.21	0.59	0.02	0.91
	Late	6.85	6.78				

^1^ CON = Conventional pearl millet; BMR = BMR-trait pearl millet. ^2^ Gen = effect of genotype; Planting = effect of planting date (early or late, 14 d apart) of pearl millet genotypes.

**Table 2:** Undigestible NDF and *in-vitro* DM digestibility of pearl millet genotypes planted at different dates (Trial 1).

		PM Genotype ^1^			*p*-Value ^2^		
Item	Planting	CON	BMR	SEM	Gen	Planting	Gen × Planting
uNDF_240_, % DM	Early	18.6	16.0	0.64	0.01	0.99	0.16
	Late	17.8	16.9				
uNDF_240_, % NDF ^3^	Early	32.6	27.8	1.0	<0.01	0.98	0.24
	Late	31.5	29.0				
pdNDF, % NDF ^4^	Early	67.4	72.2	1.0	<0.01	0.98	0.24
	Late	68.6	71.0				
IVDMD, % DM ^5^	Early	71.9	72.5	0.65	0.45	0.47	0.90
	Late	72.4	72.8				
IVTDMD, %DM ^6^	Early	79.5	79.9	0.61	0.54	0.39	0.90
	Late	80.1	80.4				

^1^ CON = Conventional pearl millet; BMR = BMR-trait pearl millet. ^2^ Gen = effect of genotype; Planting = effect of planting date (early or late, 14 d apart) of pearl millet genotypes. ^3^ uNDF240 = undegraded neutral detergent fiber (after 240 h of fermentation). ^4^ pdNDF = potentially digestible neutral detergent fiber. ^5^ IVDMD = In vitro 30 h dry matter digestibility. ^6^ IVTDMD = In vitro 30 h true dry matter digestibility.

**Table 3:** Yield and nutritional composition of pearl millet genotypes mixed or not with cowpea harvested at boot or heading maturity stages (Trial 2).

		Treatments ^1^					*p*-Value ^2^			
Item	Maturity	CON	CON + CWP	BMR	BMR + CWP	SEM	Gen	CWP	Maturity	Gen × CWP
DM yield, kg/ha	Boot	8218	7008	7571	7233	489	0.07	0.07	0.24	0.86
	Heading	8616	8367	7799	6926					
DM, %	Boot	27.8	27.7	29.3	27.5	0.97	0.65	0.04	0.02	0.72
	Heading	30.6	28.9	29.1	28.4					
Ash, % DM	Boot	13.7	13.6	13.6	13.6	0.61	0.98	0.67	<0.01	0.55
	Heading	14.9	14.9	14.6	15.3					
CP, % DM	Boot	14.8	14.7	14.5	15.8	0.60	0.19	0.02	0.35	0.89
	Heading	13.2	15.2	14.4	15.4					
aNDFom % DM	Boot	54.9	54.2	53.5	53.1	1.06	0.02	0.02	0.34	0.34
	Heading	55.0	53.6	54.4	50.0					
ADFom, % DM	Boot	36.5	36.9	34.2	34.5	0.90	0.01	0.38	0.49	0.94
	Heading	37.5	35.9	36.0	34.7					
ADL, %DM	Boot	4.84	4.80	4.14	4.18	0.32	0.05	0.70	0.34	0.25
	Heading	4.92	4.64	4.30	4.90					
ADL, % aNDFom	Boot	8.89	9.28	7.76	7.90	0.62	0.18	0.14	0.21	0.22
	Heading	8.98	8.80	8.02	0.10					
WSC, %DM	Boot	8.10	8.66	8.87	8.60	0.42	0.60	0.69	0.51	0.58
	Heading	8.95	7.87	8.17	8.52					

^1^ CON = conventional pearl millet; CON + CWP = conventional pearl millet + cowpea, BMR = BMR pearl millet; BMR + CWP = BMR pearl millet + cowpea. ^2^ Gen = effect of genotype; CWP = effect of cowpea; Maturity = effect of maturity stage (boot or heading) at harvest.

**Table 4:** Undigestible NDF and in-vitro DM digestibility of pearl millet genotypes mixed or not with cowpea and harvested at boot or heading maturity stages (Trial 2).

		Treatments ^1^					*p*-Value ^2^			
Item	Maturity	CON	CON + CWP	BMR	BMR + CWP	SEM	Gen	CWP	Maturity	Gen × CWP
uNDF_240_^3^, % DM	Boot	17.1	17.6	14.7	14.8	0.87	<0.01	0.92	<0.01	0.57
	Heading	20.8	19.5	16.7	17.1					
uNDF_240_^3^, % NDF	Boot	31.1	32.3	27.3	27.8	1.69	<0.01	0.40	<0.01	0.35
	Heading	37.6	36.2	30.5	34.0					
pdNDF ^4^, % NDF	Boot	68.9	67.7	72.8	72.2	1.69	<0.01	0.40	<0.01	0.35
	Heading	62.4	63.8	69.6	66.1					
IVDMD, % ^5^	Boot	71.3	70.2	73.2	74.3	0.90	<0.01	0.22	<0.01	0.65
	Heading	66.3	68.5	70.4	71.5					
IVTDMD, % ^6^	Boot	78.7	77.8	80.9	81.7	0.91	<0.01	0.23	<0.01	0.93
	Heading	74.1	76.2	78.4	79.3					

^1^ CON = conventional pearl millet; CON + CWP = conventional pearl millet + cowpea; BMR = BMR pearl millet; BMR + CWP = BMR pearl millet + cowpea. ^2^ Gen = effect of genotype; CWP = effect of cowpea; Maturity = effect of maturity stage (boot or heading) at harvest. ^3^ uNDF240 = undegraded neutral detergent fiber (after 240 h of fermentation). ^4^ pdNDF = potentially digestible neutral detergent fiber. ^5^ IVDMD = In vitro 30 h dry matter digestibility. ^6^ IVTDMD = In vitro 30 h true dry matter digestibility.

## Data Availability

Not applicable.

## Abbreviations

ADFom, acid detergent fiber; ADL, acid detergent lignin; BMR, brown midrib pearl millet genotype; CON, conventional pearl millet genotype; CP, crude protein; CWP, cowpea; DM, dry matter; IVDMD, in vitro dry matter digestibility; IVNDFD, in vitro neutral detergent fiber digestibility; IVTDMD, in vitro true dry matter digestibility; aNDFom, neutral detergent fiber; pdNDF, potentially digestible neutral detergent fiber; PM, pearl millet; uNDF240, undigestible neutral detergent fiber; WSC, water soluble carbohydrates.
